# Changes in neuropsychiatric symptoms and caregivers’ distress in behavioral variant frontotemporal dementia and Alzheimer’s disease in 12 months

**DOI:** 10.1192/j.eurpsy.2022.466

**Published:** 2022-09-01

**Authors:** M. Yassuda, T. Lima-Silva

**Affiliations:** University of São paulo, Gerontology, São Paulo, Brazil

**Keywords:** behavioral dementia frontotemporal (bvFTD), Alzheimer´s disease, aging, Neuropsychiatric symptoms

## Abstract

**Introduction:**

In behavioral variant frontotemporal dementia (bvFTD) neuropsychiatric symptoms are a significant concern as they impact care management and caregiver wellbeing.

**Objectives:**

To describe change in individual neuropsychiatric symptoms and associated caregivers’ distress assessed by the Neuropsychiatry Inventory (NPI) in patients diagnosed with bvFTD and Alzheimer’s disease (AD) from baseline to a 12-month follow-up.

**Methods:**

The sample consisted of 31 patients diagnosed with bvFTD and 28 patients with AD and their caregivers. The NPI and the Addenbrooke´s Cognitive Examination Revised (ACE-R) were applied. Descriptive statistics, Mann-Whitney U test, Wilcoxon test, Chi square (χ2) were used.

**Results:**

At baseline, significantly higher scores were observed for the bvFTD group for: agitation, disinhibition and eating disturbances. The latter two were also higher in the NPI Distress subdomains. At followup, there were significantly higher scores for the bvFTD group in agitation, disinhibition, eating disturbances, hallucination and irritability. For the NPI Distress subdomains, agitation, eating disturbances and hallucination scores were significantly higher for the bvFTD group.

**Conclusions:**

In 12 months, neuropsychiatric symptoms increased in both bvFTD and AD groups. However, NPI subdomain and caregiver distress scores were statistically higher among bvFTD patients at both assessment points. Neuropsychiatric symptoms may be associated with care burden in bvFTD and should be a focal point in care management decisions.

**Disclosure:**

No significant relationships.

